# Acute Kidney Injury and Drugs Prescribed for COVID-19 in Diabetes Patients: A Real-World Disproportionality Analysis

**DOI:** 10.3389/fphar.2022.833679

**Published:** 2022-03-17

**Authors:** Yu Zhou, Jianbin Li, Linyao Wang, Xinyan Zhu, Meilian Zhang, Jiaping Zheng

**Affiliations:** ^1^ Department of Clinical Pharmacy and Pharmacy Administration, School of Pharmacy, Fujian Medical University, Fuzhou, China; ^2^ Department of Ultrasound, Fujian Provincial Maternity and Child Health Hospital, Fuzhou, China; ^3^ Department of Rehabilitation Medicine, School of Health, Fujian Medical University, Fuzhou, China

**Keywords:** pharmacovigilance, acute kidney injury, COVID-19, diabetes mellitus, drug, adverse drug reaction

## Abstract

**Background**: The information is relatively scarce regarding the occurrence of drug-induced acute kidney injury (AKI) when anti-coronavirus disease 2019 (COVID-19) drugs are prescribed for patients with diabetes mellitus (DM).

**Objective**: The objective of this study was to evaluate a pharmacovigilance signal for AKI upon the use of common drugs prescribed for COVID-19 treatment, especially in patients with DM.

**Methods**: The FDA Adverse Event Reporting System (FAERS) database were used, and data from the first quarter of 2020 to the third quarter of 2021 were retrieved. A disproportionality analysis was performed to determine whether AKI was more frequently reported with anti-COVID-19 drugs compared to that with other drugs in different populations. Further, reporting odds ratios (RORs) and their 95% confidence intervals (CIs) were used to calculate disproportionality. **Results:** We identified 33,488 COVID-19 patients and 2397 COVID-19 patients with DM. AKI was the most frequent adverse drug reaction (ADR) reported in this patient population. The primary suspected drugs related to AKI in more than half of the reports (75.60%, 127/168) were four common anti-COVID-19 drugs (remdesivir, tocilizumab, hydroxychloroquine, and lopinavir/ritonavir). Compared with other drugs in the same time window, remdesivir and lopinavir/ritonavir were associated with an increased risk of AKI in all COVID-19 patients (ROR: 3.97, 95% CI: 3.51–4.50; ROR: 4.02, 95% CI: 3.11–5.19, respectively). In COVID-19 patients with DM, remdesivir was significantly associated with AKI (ROR: 5.65, 95% CI: 4.06–7.87); meanwhile, there was a new AKI signal associated with tocilizumab (ROR: 2.37, 95% CI: 1.19–4.72). After sensitivity analyses in COVID-19 patients with DM, consistent results for remdesivir were observed; however, the AKI signals for tocilizumab were unstable.

**Conclusion:** Our study confirmed the association of AKI with the usage of common anti-COVID-19 drugs (especially remdesivir and tocilizumab) in DM patients. These safety signals suggested more individualized treatments for COVID-19 patients with comorbidities. Cross-disciplinary collaborative is needed to improve current strategy of clinical treatment and develop new approaches to management.

## 1 Introduction

Acute kidney injury (AKI) frequently develops in patients with coronavirus disease 2019 (COVID-19), with the pooled incidence reaching 6.5% and the rate being higher (32.5%) among patients in the intensive care unit (Zheng et al., 2020). Diabetes mellitus (DM) is one of the most frequent comorbidities in patients with COVID-19 (Yang et al., 2020; Ng et al., 2021). An increasing number of studies have shown higher rates of hospitalization, severe pneumonia, and mortality among DM patients infected with severe acute respiratory syndrome coronavirus 2 (SARS-CoV-2) than in non-diabetes patients with COVID-19 (Huang et al., 2020; Richardson et al., 2020; Zhou et al., 2020; Longmore et al., 2021), which are related to AKI(Lim et al., 2021).

Apart from direct cytopathic effects and secondary damage resulting from an inflammatory response, AKI in patients with SARS-CoV-2 infection might occur owing to drug-induced nephrotoxicity (Zheng et al., 2020). Drug-induced nephrotoxicity has caused a substantial medical burden. Global data suggests that drug-induced nephrotoxicity in adults accounts for 19–26% of all hospitalized cases (Kwiatkowska et al., 2021). In a nationwide cross-sectional survey from Chinese population, 280,255 hospitalized cases were screened and 1,960 cases were diagnosed as hospital-acquired AKI, among which 735 cases were defined as drug-induced AKI (37.50%) (Liu et al., 2021). Therefore, further investigation of the optimal drug management in DM patients with COVID-19 is warranted.

Emerging data highlighted AKI related to some drugs prescribed for COVID-19, which could be life-threatening or fatal, such as remdesivir (Chouchana et al., 2021; Gérard et al., 2021; Singh and Kamath 2021) and lopinavir/ritonavir (Binois et al., 2020). Assessment of AKI associated with these drugs in COVID-19 patients with DM is necessary and challenging. Global repositories of postmarketing safety reports provide an opportunity to enhance our understanding of the nephrotoxicity of the drugs prescribed for COVID-19 treatment in a real-world setting. By using large self-reporting databases, some pharmacovigilance studies have been assessed the nephrotoxicity spectrum of remdesivir (Gérard et al., 2021; Singh and Kamath 2021).

Therefore, in this study, we aimed to assess the association of AKI with the usage of common anti-COVID-19 drugs (remdesivir, tocilizumab, hydroxychloroquine, and lopinavir/ritonavir) COVID-19 patients with DM by using the FDA Adverse Event Reporting System (FAERS). To this end, we conducted a three-step analysis. First, we compared the occurrence of adverse drug reactions (ADRs) in COVID-19 patients with DM and that in all COVID-19 patients. Second, we compared relevant information from reports associated with AKI in COVID-19 patients with DM and that in all COVID-19 patients, including demographic, administrative, and ADR information. Finally, we performed a disproportionality analysis to assess the potential increased risk of AKI due to drugs prescribed for COVID-19 treatment among COVID-19 patients with DM or all COVID-19 patients.

## 2 Materials and Methods

### 2.1 Data Source

This pharmacovigilance study was conducted using the FAERS database. This database contains reports of adverse events from physicians, pharmacists, other healthcare professionals, and non-healthcare professionals, and is updated quarterly. It allows for the signal detection and quantification of the association between drugs and reporting of adverse events. In FAERS, the reporter may indicate specifically the role of the drug in the occurrence of an adverse event as a “primary suspect” (PS), “secondary suspect” (SS), or “interacting” or “concomitant” (C). ADRs and indications are coded by the FDA (U.S. Food and Drug Administration) by using Medical Dictionary for Regulatory Activities (MedDRA^®^) terminology in levels of preferred terms (PTs) and above, which identify a given sign/symptom. Since the FAERS is a publicly available and anonymized database, institutional review board approval and the requirement of informed consent were waived.

### 2.2 Report Selection

To increase the sensitivity of subsequent quantitative analyses and reduce the confounding of related disease background, a population‐focused study (Rocca et al., 2021) was performed in some subsets of the FAERS database containing COVID‐19 and DM reports between the first quarter of 2020 (2020q1) and the third quarter of 2021 (2021q3), which included at least one drug prescribed for COVID‐19 among “PS” drugs.

COVID‐19 reports were selected based on the PTs containing “COVID‐19” or “SARS‐Cov‐2” by retrieving the indication table in FAERS. Diabetes patients were identified based on the indications related to DM and the use of hypoglycemic drugs. The indications related to DM include the high level group term (HLGT) “Diabetic complications” (MedDRA 24.0 code: 10012653) and the high level term (HLT) “Diabetes mellitus (incl subtypes)” (MedDRA 24.0 code: 10012602). Hypoglycemic drugs in the drug table of FAERS were defined based on the active substances at the second level (code: A10) corresponding to the Anatomical Therapeutic Chemical (ATC) classification.

The drugs prescribed for COVID-19 in this study included remdesivir, tocilizumab, hydroxychloroquine, and lopinavir/ritonavir. Since the FAERS does not use a uniform coding system for medications, brand names and generic names (listed in the Drugs@FDA Database [www.accessdata.fda.gov/scripts/cder/daf/]) were used to identify relevant records. Essential information from each report was extracted and analyzed, including demographic data (age and sex), administrative information (type of reporter and reporting country), and ADR data (time-to-onset, drug roles, and outcome of event). Time-to-onset (i.e., latency in the occurrence of a given ADR, expressed in days) was calculated as the difference between the date the event occurred and the start of therapy.

### 2.3 Disproportionality Analysis

Disproportionality analysis was conducted to detect increased reporting of drugs prescribed for COVID-19 associated AKI compared to all other reports in the database during the same time period. Reporting odds ratios (ROR) with 95% confidence intervals (CIs) were calculated to determine disproportionality (Noguchi et al., 2021). The ROR was deemed significant if the lower limit of the 95% CI was >1. To evaluate the robustness of our main analyses, five sensitivity analyses were conducted among COVID-19 patients with DM.

Model 1: Exclusion of hypertension reports; hypertension patients were identified based on the indications related to hypertension (MedDRA 24.0 code of HLT: 10000356, 10052741, 10010164, 10036557, 10038465, 10020774) and use of hypotensive drugs (ATC code: C02).

Model 2: Restricted to reports with severe outcomes of an event. In the FAERS database, a serious outcome of an event is defined as one of the following: death, life-threatening, hospitalization-initial or prolonged, disability requiring intervention to prevent permanent impairment/damage, congenital anomaly, or other serious events.

Model 3: Exclusion of reports listing angiotensin-converting enzyme inhibitors (ACEIs) or angiotensin receptor blockers (ARBs).

Model 4: Exclusion of reports listing dipeptidyl peptidase 4 (DPP4) inhibitors.

Model 5: Exclusion of known reports listing nephrotoxic drugs (e.g., vancomycin, bumetanide, chlorothiazide, spironolactone, hydrochlorothiazide, aciclovir, amikacin, amphotericin b, chlortalidone, and nimesulide).

Data processing and analyses were conducted using R 4.0.2 (R Institute for Statistical Computing, Vienna, Austria).

## 3 Results

### 3.1 Ranking of Adverse Drug Reactions

**FIGURE 1:**
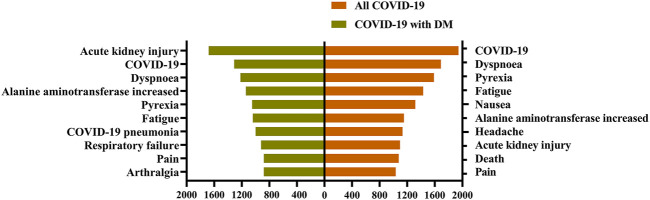
Ranking of adverse drug reactions in different patients. Adverse drug reactions are visualized on the *Y*-axis, their occurrence number are shown on the *X*-axis. COVID-19: coronavirus disease 2019; DM: diabetes mellitus. A report may have one or more drug adverse reactions.

Between 2020q1 and 2021q3, we identified 33,488 COVID-19 patients and 2397 COVID-19 patients with DM. Figure 1 shows the ranking of ADRs in COVID-19 patients with DM and all COVID-19 patients. In the ADR rankings of all COVID-19 patients, AKI was eighth on the list; however, among COVID-19 patients with DM, AKI was the most frequent ADR.

### 3.2 Descriptive Analyses

We further analyzed the characteristics of patients who experienced AKI **(** Table 1). Of these, 168 cases were reported among COVID-19 patients with DM, and 1,096 cases involved COVID-19 patients overall. The median age in these cases was 66 (range, 60–72) and 65 (range, 54–73) years, respectively. More men than women tended to report AKI among COVID-19 patients with DM and COVID-19 patients overall (60.71% vs. 35.12%; 61.22% vs. 27.92%). COVID-19 patients with DM had a shorter median time-to-onset of AKI (2 days; Q1–Q3: 1–6) in comparison with all COVID-19 patients (3 days; 1–6). Among COVID-19 patients with DM and all COVID-19 patients, cases associated with AKI were reported primarily by non-healthcare professionals (95.24% vs. 90.24%, respectively), and the vast majority were from the United States (73.21% vs. 65.05%, respectively). In the assessment of outcomes, COVID-19 patients with DM tended to report death, life-threatening disease, hospitalization (initial or prolonged), and other serious events more frequently than all COVID-19 patients (33.74% vs. 28.47%, 27.38% vs. 20.16%, 41.67% vs. 45.71%, and 66.07% vs. 62.86%, respectively). More notably, the drugs prescribed for COVID-19 (remdesivir, tocilizumab, hydroxychloroquine, and lopinavir/ritonavir) accounted for more than half of corresponding cases (75.60%, 127/168) as “PS” drugs induced AKI.

**TABLE 1:** Characteristics of patients who experienced AKI.

	COVID-19 with DM (*n* = 168)	All COVID-19 (*n* = 1,096)
Age, years, median (Q1–Q3)	66 (60–72)	65 (54–73)
Sex, number (%)		
Female	59 (35.12)	306 (27.92)
Male	102 (60.71)	671 (61.22)
Not reported	7 (4.17)	119 (10.86)
Type of reporter, number (%)		
Health professional	160 (95.24)	989 (90.24)
Non-health professional	7 (4.17)	49 (4.47)
Unknown	1 (0.60)	58 (5.29)
Reporting country, number (%)		
First	US/123 (73.21)	US/713 (65.05)
Second	FR/10 (5.95)	FR/74 (6.75)
Third	BR/9 (5.34)	ES/68 (6.20)
Time-to-onset, days, IQR (Q1–Q3)	2 (1–6)	3 (1–6)
Outcome of event^a^, number (%)		
Death	55 (32.74)	312 (28.47)
Life-threatening	46 (27.38)	221 (20.16)
Hospitalization (initial or prolonged)	70 (41.67)	501 (45.71)
Disability	6 (3.58)	31 (2.83)
Required intervention to prevent permanent impairment/damage	1 (0.60)	14 (1.28)
Congenital anomaly	0	6 (0.55)
Other serious events	111 (66.07)	689 (62.86)
“PS” drugs prescribed for COVID-19 involved, number (%)		
Remdesivir	109 (64.88)	444 (40.51)
Tocilizumab	10 (5.95)	38 (3.47)
Hydroxychloroquine	8 (4.76)	117 (10.68)
Lopinavir/Ritonavir	0	70 (6.39)

AKI: acute kidney injury; COVID-19: coronavirus disease 2019; DM: diabetes mellitus; IQR: interquartile range; PS: primary suspect; US: united states; FR: france; BR: brazil; ES: espana.

a A report may have one or more outcomes.

### 3.3 Reporting Risk Differences of Drugs Prescribed for COVID-19

Given the high number of cases associated with AKI after exposure to the drugs prescribed for COVID-19, we quantitatively assessed a potential risk of reporting AKI with the drugs prescribed in different patients by performing a disproportionality analysis (Table 2). In comparison with the other drugs in the same time window, the use of remdesivir and lopinavir/ritonavir was associated with an increased risk of reporting AKI in all COVID-19 patients (ROR: 3.97, 95% CI: 3.51–4.50; ROR: 4.02, 95% CI: 3.11–5.19). In COVID-19 patients with DM, the association with remdesivir remained significant (ROR: 5.65, 95% CI: 4.06–7.87). Meanwhile, there was a new AKI signal associated with tocilizumab (ROR: 2.37, 95% CI: 1.19–4.72). However, for COVID-19 patients with DM and all COVID-19 patients, reporting risk associated with AKI for hydroxychloroquine was not found.

**TABLE 2:** Disproportionality analyses for AKI upon the use of drugs prescribed for COVID-19.

As “PS” drugs	COVID-19 with DM (n = 168)		All COVID-19 (n = 1,096)	
	Cases	ROR (95% CI)	Cases	ROR (95% CI)
Remdesivir	109	5.65 (4.06–7.87)^*^	444	3.97 (3.51–4.50)^*^
Tocilizumab	10	2.37 (1.19–4.72)^*^	38	0.66 (0.48–0.92)
Hydroxychloroquine	8	1.20 (0.57–2.52)	117	1.08 (0.89–1.31)
Lopinavir/Ritonavir	0	N/D	70	4.02 (3.11–5.19)^*^

The asterisk indicates statistical significance.

AKI: acute kidney injury; COVID-19: coronavirus disease 2019; DM: diabetes mellitus; PS: primary suspect; ROR: reporting odds ratio; CI: confidence interval; N/D, not determined.

Five kinds of sensitivity analysis were performed based on factors such as concomitant diseases and drugs **(** Table 3). Consistent results were observed in their sensitivity analyses of remdesivir. However, the RORs only remained significant in model 2 (ROR: 2.31, 95% CI: 1.15–4.62) and model 4 (ROR: 2.42, 95% CI: 1.16–5.05).

**TABLE 3:** Sensitivity analyses of reporting risks for AKI upon the use of drugs prescribed for COVID-19 in DM.

	Remdesivir		Tocilizumab		Hydroxychloroquine	
	Cases	ROR (95%CI)	Cases	ROR (95%CI)	Cases	ROR (95%CI)
Model 1	90	9.20 (5.87–14.42)^*^	4	1.39 (0.49–3.95)	4	0.89 (0.32–2.49)
Model 2	102	5.47 (3.90–7.66)^*^	10	2.31 (1.15–4.62)^*^	8	1.16 (0.55–2.44)
Model 3	86	8.12 (5.09–12.95)^*^	5	1.44 (0.56–3.71)	8	1.89 (0.88–4.08)
Model 4	101	6.76 (4.59–9.96)^*^	9	2.42 (1.16–5.05)^*^	5	0.83 (0.33–2.10)
Model 5	62	5.07 (3.38–7.62)^*^	5	2.58 (0.99–6.74)	8	1.81 (0.85–3.85)

The asterisk indicates statistical significance.

AKI: acute kidney injury; DM: diabetes mellitus; ROR: reporting odds ratio; CI: confidence interval.

Model 1: excluding hypertensive reports.

Model 2: restricted to reports with severe outcomes of an event.

Model 3: excluding reports listing angiotensin-converting enzyme inhibitors (ACEIs) or angiotensin receptor blockers (ARBs).

Model 4: excluding reports listing dipeptidyl peptidase 4 (DPP4) inhibitors.

Model 5: excluding reports listing known nephrotoxic drugs (vancomycin, bumetanide, chlorothiazide, spironolactone, hydrochlorothiazide, aciclovir, amikacin, amphotericin b, chlortalidone, nimesulide).

## 4 Discussion

In this pharmacovigilance study, we found that AKI is the most frequent ADR in COVID-19 patients with DM. Moreover, the “PS” drugs related to AKI in more than half of reports were the main drugs prescribed for COVID-19 (remdesivir, tocilizumab, hydroxychloroquine, and lopinavir/ritonavir). In addition, disproportionality analysis suggested an increased risk of reporting AKI with remdesivir and tocilizumab than with other drugs in COVID-19 patients with DM. These results will raise awareness about the potential signals regarding the risk of drug-induced AKI in COVID-19 patients with DM.

During the development phase of remdesivir, kidney injuries were observed in murine and mammalian models. AKI also has been frequently reported in clinical trials and case series (Dubert et al., 2020; Spinner et al., 2020). Three subsequent postmarketing real-world studies reported that remdesivir was associated with an increased risk of reporting AKI (Chouchana et al., 2021; Gérard et al., 2021; Singh and Kamath 2021). Our study supports these results, and the association between remdesivir and AKI remained significant in COVID-19 patients with DM. Of note, lopinavir/ritonavir and tocilizumab therapy is not generally considered nephrotoxic. However, in our study, lopinavir/ritonavir and tocilizumab produced a disproportionality signal of AKI in COVID-19 patients with and without DM. These signals may be related to multilateral relationships among infection, disease, and drugs.

The interconnections among DM, COVID-19, and drugs may not be as simple as they appear. DM is currently considered a risk factor for increased COVID-19 severity and worse outcomes, including higher mortality (Lim et al., 2021). DM and SARS-CoV-2 infection have shared pathogenic pathways, which has therapeutic implications (Drucker 2020). Angiotensin-converting enzyme 2 (ACE2), a part of the renin–angiotensin–aldosterone system (RAAS) that is highly expressed in the kidney, is the main entry receptor for SARS-CoV-2 (Lim et al., 2021). Routine use of ACEIs and ARBs for chronic conditions upregulates ACE2 expression (Huang et al., 2020). The evidence shows that the use of ACEIs/ARBs was not associated with an increased risk of all-cause mortality, but might increase the risk of AKI in severe COVID-19 patients (Akhtar et al., 2020). Dipeptidyl peptidase 4 (DPP4) is well recognized to have an important function in glucose homeostasis and may also act as a binding target for SARS-CoV-2 (Lim et al., 2021). Current evidence does not indicate safety issues associated with the use of DPP4 inhibitors in COVID-19 patients with DM(Cariou et al., 2020; Iacobellis 2020). However, the effects of DPP4 inhibition on the immune response in patients with diabetes is a matter of debate (Iacobellis 2020). The potential effects of DPP4 inhibitors on the susceptibility or severity of SARS-CoV-2 infection needs to be studied in future trials. In addition, diabetic patients have been shown to have an elevated pro-inflammatory cytokine level, especially interleukin-1 (IL-1), interleukin-6 (IL-6) and tumor necrosis factor-α (TNF-α) (Huang et al., 2020). The low-grade chronic inflammatory state can favor the aggravation of an inflammatory response induced by SARS-CoV-2 (Lim et al., 2021). Thus, if cytokine storms in COVID-19 spread to the kidney, AKI will inevitably occur.

In summary, the occurrence of AKI should be multifactorial in COVID-19 patients with DM, which increases the difficulty of assessment of drug-induced AKI. In this study, we conducted five sensitivity analyses of COVID-19 patients with DM to evaluate the robustness of our main analyses. Diabetes patients often show accompanying hypertension, which can also cause potential damage to the kidney. In model 1, we excluded the influence of hypertensive factors. COVID-19 patients from the intensive care unit have a higher pooled incidence of AKI than those in the general ward (Zheng et al., 2020). In our data, patients with serious outcomes accounted for the vast majority (89.03%, 2134/2397). In model 2, we excluded the influence of disease severity by restricting to reports showing severe outcomes of an event. As was stated above, except for nephrotoxic drugs, the use of ACEIs, ARBs, and DPP4 inhibitors may have interfered with our analysis. Finally, in model 3 to model 5, we excluded the influence of concomitant drugs leading to potential AKI in COVID-19 patients. These sensitivity analyses of remdesivir showed similar results. However, the AKI signals of tocilizumab were unstable due to the influence of concomitant diseases and drugs. Whether tocilizumab in COVID-19 with DM patients increases the susceptibility of AKI needs to be confirmed by clinical studies.

Our study had several limitations. The primary limitations related to the FAERS database are well-known. First, the media attention on COVID-19 and the recent publication of ADRs in the literature may have affected reporting behaviors. Second, the FAERS database provides limited patient characteristic data. Missing history for kidney disease and treatment with nephrotoxic drugs made the causality assessment difficult. Our current data only suggests that these drugs can increase the reporting risk of AKI in COVID-19 patients with DM (Montastruc et al., 2011). Third, the MedDRA definition of AKI in the FAERS database is simple and lacks relevant inspection information. AKI from acute kidney disease or and chronic kidney disease might not be differentiated by the reporter. Finally, in addition to considering the effects of known nephrotoxic drugs, drug-drug interactions leading to potential AKI need to be further explored in COVID-19 patients with DM. For example, a recent study showed that treatment with lopinavir/ritonavir and hydroxychloroquine is associated with an increase in the incidence of AKI in non-ICU COVID-19 patients (Schneider et al., 2021). Despite these limitations, FAERS contains large real-world data from various populations and is suitable for discovering a new correlation between drugs and AKI.

Our study provides evidence supporting the potential association of AKI with the usage of common anti-COVID-19 drugs (especially remdesivir and tocilizumab) in DM patients, and emphasizes the importance of more individualized treatments for COVID-19 patients with comorbidities. In the future, cross-disciplinary collaborative is needed to improve current strategy of clinical treatment and develop new approaches to management.

## Data Availability

Publicly available datasets were analyzed in this study. This data can be found here: https://fis.fda.gov/extensions/FPD-QDE-FAERS/FPD-QDE-FAERS.html.
